# Low Birthweight Is Associated with Higher Risk of High Blood Pressure in Chinese Girls: Results from a National Cross-Sectional Study in China

**DOI:** 10.3390/ijerph16162898

**Published:** 2019-08-13

**Authors:** Xijie Wang, Yanhui Dong, Zhiyong Zou, Jun Ma, Zhaogeng Yang, Di Gao, Yanhui Li, Minh Thien Nguyen

**Affiliations:** 1 Institute of Child and Adolescent Health, School of Public Health, Peking University, Beijing 100191, China; 2 Key laboratory of Reproductive Health, National Health Commission of the People’s Republic of China, Beijing 100191, China; 3 Murdoch Children’s Research Institute, Flemington Road, Parkville, Melbourne 3052, Australia; 4 Department of Paediatrics, University of Melbourne, Parkville, Melbourne 3052, Australia

**Keywords:** low birthweight, hypertension, weight change, adolescent health

## Abstract

Objective: To investigate the relationship between low birthweight (LBW) and blood pressure and to assess whether LBW leads to a higher risk of high blood pressure (HBP) by gender in Chinese students aged 6–18 years. Also, to investigate whether the association was affected by childhood obesity. Methods: Data was obtained from a baseline dataset of a national school-based program. Anthropometric parameters, including height, weight, and blood pressure, were measured, while birthweight and other characteristics were obtained from questionnaires. Stratified chi-squared tests were used to compare the prevalence of HBP between LBW and normal birthweight (NBW) groups in each age and sex category. Multivariable logistic regressions were conducted to estimate the HBP risks in each birthweight group. Results: Both systolic and diastolic blood pressure showed a U-shaped relationship with increased birthweight. Compared to NBW groups, LBW girls showed a higher HBP risk, with an odds ratio of 1.29 (95% confidence interval (CI): 1.02, 1.64, *p* = 0.033), regardless of their current body mass index status, while no significant association in boys was found. **Conclusions**: Low birthweight is associated with higher HBP risk in adolescent girls, regardless of their childhood BMI status.

## 1. Introduction

Hypertension, or high blood pressure (HBP), is one of the largest contributors to cardiovascular disease disability-adjusted life-years (DALYs) [1]. In China, 6.4% of school-aged children have HBP, and a considerable proportion of these children will develop hypertension in adulthood [2,3].

Previous studies indicate that low birthweight (LBW), defined as a birthweight less than 2500 g, is an important indicator of intrauterine growth status and a predictor of adverse health outcomes, such as hypertension and cardio-metabolic diseases throughout life [4,5,6,7]. For instance, extremely low birthweight has been shown to lead to severe hypertension [8]. However, some studies have showed little effect of LBW on blood pressure in young adults [9,10]. On the other hand, some recent studies suggest that the co-existence of low birthweight and later obesity, rather than low birthweight itself, was a strong risk factor of HBP during adolescence [11,12,13,14,15,16,17]. The relationship between LWB and HBP remains unclear, and obesity during school-age is seldom brought into consideration. It is still controversial to suggest that LBW could be associated with childhood HBP risk and if the association could be influenced by obesity.

Using baseline data from a national school program in China, the present study aimed to evaluate the relationship of LBW with blood pressure and assess whether LBW leads to a higher HBP risk by gender in Chinese students aged 6–18 years.

## 2. Materials and Methods

### 2.1. Study Setting and Participants’ Engagement

Data was obtained from the baseline data of a national school program in 2013. The sampling procedure of this study has been published previously in detail [18]. Briefly, the original study was a national school program involving around 70,000 participants from seven provinces in China, including Liaoning, Tianjin, Ningxia, Shanghai, Chongqing, Hunan, and Guangdong. In each province, at least 10,000 participants from 12 to 16 primary and secondary schools were randomly selected to participate. Altogether, 45,319 children aged 6–18 were included in the present analysis after excluding children with missing values on height (*n* = 1414), birthweight (*n* = 12,475), systolic blood pressure (SBP; *n* = 209), diastolic blood pressure (DBP; *n* = 37), and children with a birthweight over 4000 g (*n* = 5893). The study was approved by the Peking University Ethics Committee (No. IRB0000105213034). All students participated and their parents signed informed consent forms.

### 2.2. Birthweight Data

Birthweight data was collected using a standard parent questionnaire. Parents were required to record their children’s birthweight based on the record given by their birth certificate or by their health clinic. If they did not have one, they were asked to recall the birthweight based on measurements recorded themselves. About 70.9% of the parents of participants recorded the information of birthweight based on a health clinic card or birth certificate.

### 2.3. Anthropometric Measurements

All participants underwent a complete anthropometric evaluation according to a standardized study protocol [18]. Height was measured two times using a portable stadiometer (model TZG, China) to the nearest 0.1 cm, with the students standing upright and barefoot. Weight was measured with a lever-type weight scale (model RGT-140, China) to the nearest 0.1 kg, with students wearing no shoes and light clothes only. The average of two repeats were calculated for height and weight.

### 2.4. Blood Pressure Measurement

Blood pressure was measured according to the recommendations set out by the National High Blood Pressure Education Program (NHBPEP) Working Group in Children and Adolescents [19], using a mercury sphygmomanometer (model XJ11D, China) and a stethoscope (model TZ-1, China) from the right arm with an appropriate cuff size. Students were seated comfortably for at least 5 min prior to the first reading. SBP was determined by the onset of the first Korotkoff sound and DBP was determined by the fifth Korotkoff sound. Blood pressure was measured twice with a 1 min gap between replicates. The averages of SBP and DBP were calculated. All anthropometric measurements were rechecked in 5% of subjects daily. If the proportion of invalid cases exceeded 10%, all the measures of that day were considered as invalid and were measured again.

### 2.5. Measurements Classifications

LBW was defined as a birthweight less than 2500 g, according to World Health Organization [5]. Normal birthweight was defined as a birthweight between 2500 g to 4000 g. Current body mass index (BMI) indicated the children’s BMI at the time of study recruitment, and the children were divided into two groups (normal/underweight versus overweight/obese) according to the reference for Chinese children and adolescents [20]. High blood pressure was defined as systolic blood pressure or diastolic blood pressure higher than the 95th age-, sex-, and height- specific percentile according to the reference of the NHBPEP working group [19].

### 2.6. Questionnaire Data Collection

The questionnaire of this survey was developed with reference to the Chinese National Survey on Students Constitution and Health (CNSSCH), which was conducted every 5 years in 31 provinces of China. Data collected via questionnaire included long term residence (urban/rural, for the past year), daily physical activity time, family history of hypertension (yes/no), only child of the family (yes/no), and breastfed for over a month (yes/no).

Physical activity included vigorous-intensity and moderate-intensity. Vigorous-intensity physical activities were described as aerobic activities that significantly increase heart rate and breathing, for example, running, basketball, football, and swimming, while moderate-intensity physical activities were described as aerobic activities that increase heart rate and breathing to some extent, for example, cycling, table tennis, badminton, and calisthenics. Children reported their activity frequency (days over the past 7 days) and duration (hours and minutes in each of those days) on an activity card. Daily physical activity time was calculated as average daily time = (days × (time in each of those days))/7.

Before the survey, all eligible investigators were involved in a training session to get familiar with the whole process. 3% of all participants were asked to refill the questionnaires within a week for recheck. All questionnaires were checked for logical and integral accuracy by the time of data input.

### 2.7. Statistical Analysis

Continuous variables were reported as means with standard deviation and categorical variables reported as numbers with percentages. The quadratic regression model was used to describe the association between birthweight and blood pressure values [21]. The differences between normal birthweight (NBW) and LBW group were tested with independent sample *t*-tests or chi-squared tests. Age-stratified chi-squared tests were conducted to compare the prevalence of HBP between the two birthweight groups. Sex-specific multivariable logistic regression models were used to compare HBP risk in different birthweight groups, as well as in BMI-stratified birthweight groups. Three models were conducted: model 1 was adjusted for age and province; model 2 was further adjusted for height, urban/rural living condition, daily physical activity (hours), family history of hypertension (yes/no), only child status (yes/no), and breastfeeding status (yes/no); model 3 was further adjusted for current BMI. All analyses were performed using Stata 14.0 (StataCorp, College Station, TX, USA) and were two-sided with *p* < 0.05 considered as significant.

## 3. Results

Among the 45,319 children, 43,220 (95.4%) were NBW, while 2099 (4.6%) were LBW. The current BMI of NBW and LBW was 18.4 kg/m^2^ and 17.9 kg/m^2^ (*p* < 0.001), respectively. Other participant characteristics, including anthropometric and demographic features by birthweight groups, are shown in Table 1. The differences between the two birthweight groups were all statistically significant.

**Figure 1:**
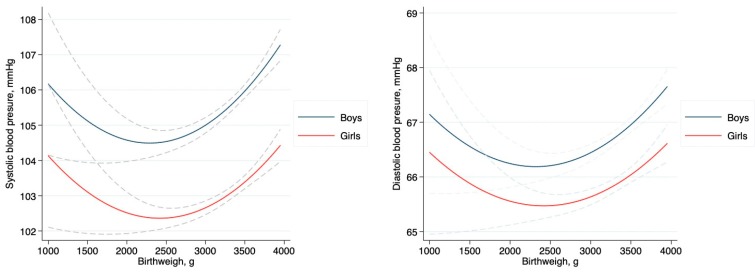
Change of systolic and diastolic blood pressure along with increased birthweight in Chinese children aged 6–18, by sex.

Figure 1 shows the relationship between SBP, DBP, and increased birthweight by sex. U-shaped relationships were observed from both SBP and DBP change in both sexes. Both BP levels showed a decreased trend while in the LBW category, and showed increased trends in the NBW category.

In total, 9.27% of LBW boys and 9.49% of LBW girls, versus 9.07% of NBW boys and 8.38% NBW girls had HBP, as is displayed in Table 2. Generally, the HBP prevalence was higher in the LBW group in both sexes, though no statistically significant difference was found.

Table 3 presented the results of logistic regression for HBP risk in LBW children, compared to NBW children (i.e., the reference group). In the fully-adjusted model, where children’s age, current BMI, height, living condition (urban/rural), and daily physical activity time were adjusted, LBW girls showed a 1.29 (95% confidence interval (CI): 1.02, 1.64) chance of HBP risk compared with the NBW group (*p* = 0.033). Meanwhile, no statistically significant differences were observed in boys.

**Figure 2:**
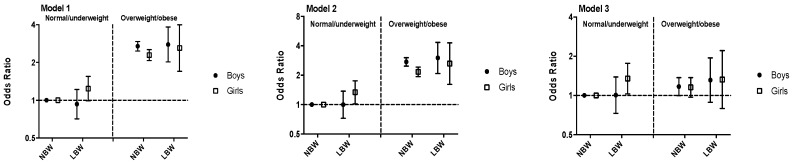
Odds ratios and 95% CIs for high blood pressure of different birthweight and current BMI categories among Chinese children. NBW, normal birthweight; LBW, low birthweight. Model 1 was adjusted for age and province; model 2 was further adjusted for height, urban/rural living condition, daily physical activity, family history of hypertension (yes/no), only child status (yes/no), and breast feeding (if or not); model 3 was further adjusted for current BMI.

When children were further stratified according to their current BMI, LBW girls also showed higher risks of HBP than NBW girls, regardless of their current BMI status. For example, in the fully-adjusted model, odds ratios for HBP in LBW groups were 1.35 (95% CI: 1.02, 1.76, *p* = 0.030) in normal/underweight girls, and 1.33 (95% CI: 0.80, 2.21, *p* = 0.280) in overweight/obese girls, with NBW and normal/underweight girls set as a reference. However, LBW did not increase the risk of HBP in normal/underweight boys. Results for the stratified logistic regression are displayed in Figure 2.

## 4. Discussion

HBP in childhood not only predisposes people to hypertension in adulthood [22], it is also associated with an increased risk of an early set of cardiovascular disease and mortality [23]. Thus, screening and identification of high-risk children for potential early intervention are of high priority. In this study, with cross-sectional data from a Chinese nationwide survey, we found a U-shaped relationship between birthweight and blood pressure levels, that is, compared to those with normal birthweight, children and adolescents with lower or higher birthweight had relatively higher levels of SBP and DBP. However, the increased HBP risk only existed in low birthweight girls, no matter whether they were obese or not.

It has always been of interest to explore the association between LBW and later HBP, and the results of previous studies are varied. One study conducted in Jerusalem found that LBW had little influence on blood pressure in adolescents aged 17 [16], while another study found being born small for one’s gestational age may be a predictor of raised blood pressure when an individual is in their early 30s [24]. Both conclusions were supported by numbers found in later studies [25,26,27,28]. Beside the single effect of birthweight, some previous studies also concluded that the low-to-high postnatal weight gain pattern could positively relate to unfavorable blood pressure levels in early adolescence and adulthood [10,13,17,27]. However, children’s BMI status at school age were seldom mentioned, nor were the sex disparities. According to results of the present study, we assume that in childhood HBP development, current BMI status plays a more important role in boys, while birthweight relative to weight gain progress more significantly affects girls.

HBP development depends a lot on one’s personal hormone milieu, endothermic system, age, and age-related adiposity accumulation, which are all sex-specific [28,29] and may therefore lead to the outcome of different programming in male and female adolescents. Though this inference may need further verification, the results may lead to changes in the present universal screening on childhood HBP, [30,31] as evidence-based targeted screening is actually more cost-effective for developing countries and areas [32]. Instead, a more comprehensive guidance and management of maternal nutrition status, which keeps children healthy from birth, should be more emphasized.

The first limitation of our study is the cross-sectional design, from which we cannot conclude into a causal relationship. However, as the identification of LBW exists before childhood HBP, a causal relationship between LBW and childhood HBP is quite reasonable. Secondly, there were 17.5% of children from the present analysis who were excluded from the present research for missing birthweight data, and this may cause analysis bias. Thus, the results of our study are still in need of further verification in more representative populations. Furthermore, to assist comparisons with previous studies, the present study conducted measurements based on the 2004 version of the NHBPEP working group [19]. Although there are those saying that children’s cardiovascular risk may have been underestimated with the 2004 standard [33,34], our team has found little difference in assessing the influence of birthweight on HBP [35]. The influence of HBP standard might be very little.

## 5. Conclusions

Low birthweight is associated with a higher prevalence of HBP in girls. Normal/underweight girls are of the same risk of HBP as overweight/obese girls, and thus it is always of great importance to maintain the best nutrition status during pregnancy.

## Figures and Tables

**Table 1:** Demographic characteristics of Chinese children in moderate and low birthweight groups.

Variable	Normal Birthweight	Low Birthweight	*p*-value
	*n* = 43,220	*n* = 2099	
Male, *n* (%)	21,377 (49.46%)	1014 (48.31%)	
Age (year)	10.6 ± 3.3	10.4 ± 3.3	0.020
Birthweight (g)	3263.3 ± 356.4	2050.6 ± 333.3	<0.001
Height (cm)	144.6 ± 16.8	142.2 ± 17.0	<0.001
Weight (kg)	40.0 ± 15.3	37.6 ± 14.3	<0.001
Current BMI (kg/m^2^)	18.4 ± 3.7	17.9 ± 3.5	<0.001
BMI group, *n* (%)			
Normal/underweight	33,212 (76.84%)	1709 (81.42%)	<0.001
Overweight/obese	10,008 (23.16%)	390 (18.58%)	<0.001
Systolic blood pressure (mmHg)	104.3 ± 12.1	103.3 ± 12.4	<0.001
Diastolic blood pressure (mmHg)	66.3 ± 8.8	65.6 ± 9.2	<0.001
Urban residence, *n* (%)	26,793 (61.99%)	1463 (69.70%)	<0.001
Daily physical activity (hours/day)	3.2 ± 2.6	3.6 ± 2.6	<0.001
Family history of hypertension, *n* (%)	19,202 (52.15%)	736 (49.20%)	0.025
The only child of the family, *n* (%)	30,791 (71.24%)	1443 (68.75%)	0.014
Breastfeeding ≥ 1 month, *n* (%)	31,502 (84.61%)	1159 (76.20%)	<0.001

Notes: the *p*-values between the two birthweight groups were calculated using a *t*-test for continuous variables and χ^2^ test for categorical variables; abbreviations: BMI (body mass index).

**Table 2:** Gender- and age-specific prevalence of high blood pressure stratified by birthweight and sex, N (%).

Gender	Age Group	Normal Birthweight		Low Birthweight		*p*-value
		Number of Observations	HBP Prevalence, N (%)	Number of Observations	HBP Prevalence, N (%)	
Boys	6–8	7269	626 (8.61)	412	35 (8.50)	0.934
	9–11	5877	720 (12.25)	279	28 (10.04)	0.268
	12–15	4816	397 (8.24)	182	21 (11.54)	0.115
	16–18	3415	196 (5.74)	141	10 (7.09)	0.500
	Total	21,037	1939 (9.07)	1014	94 (9.27)	0.829
Girls	6–8	7096	645 (9.09)	331	32 (9.67)	0.721
	9–11	5389	702 (13.03)	263	37 (14.07)	0.625
	12–15	5155	373 (7.24)	273	27 (9.89)	0.102
	16–18	4203	110 (2.62)	218	7 (3.21)	0.594
	Total	21,843	1830 (8.38)	1085	103 (9.49)	0.197

Notes: HBP, high blood pressure. Data for HBP prevalence was displayed as numbers and row percentages of subjects with HBP in each age, sex, and birthweight groups.

**Table 3:** Odds ratios of high blood pressure in the low birth weight group compared with the normal birth weight group.

Sex	Model 1		Model 2		Model 3	
	OR (95% CI)	*p-*value	OR (95% CI)	*p-*value	OR (95% CI)	*p*-value
Total (*n* = 45,319)						
NBW	1 (Reference)		1 (Reference)		1 (Reference)	
LBW	1.02 (0.89, 1.18)	0.743	1.14 (0.97, 1.35)	0.119	1.16 (0.98, 1.37)	0.087
Boys (*n* = 22,391)						
NBW	1 (Reference)		1 (Reference)		1 (Reference)	
LBW	0.93 (0.76, 1.14)	0.468	1.03 (0.81, 1.31)	0.809	1.05 (0.83, 1.34)	0.672
Girls (*n* = 22,928)						
NBW	1 (Reference)		1 (Reference)		1 (Reference)	
LBW	1.16 (0.95, 1.41)	0.151	1.27 (1.01, 1.61)	**0.045**	1.29 (1.02, 1.64)	**0.033**

Notes: NBW, normal birth weight; LBW, low birth weight; OR, odds ratio; CI, confidence interval. Bold values in the table indicate to statistically significant *P* value. Model 1 was adjusted for age and province; model 2 was further adjusted for height, urban/rural living condition, daily physical activity (hours), family history of hypertension (yes/no), only child status (yes/no), and breast feeding (yes/no); model 3 was further adjusted for current body mass index.

## Abbreviations

HBP	high blood pressure
SBP	systolic blood pressure
DBP	diastolic blood pressure
BMI	body mass index
LBW	low birthweight
NBW	normal birthweight
NHBPEP	National High Blood Pressure Education Program
